# The effect of physical exercise during competitions and in simulated conditions on hormonal-neurophysiological relationships in kickboxers

**DOI:** 10.5114/biolsport.2024.133662

**Published:** 2023-12-21

**Authors:** Łukasz Rydzik, Zbigniew Obmiński, Wojciech Wąsacz, Marta Kopańska, Rafał Kubacki, Małgorzata Bagińska, Łukasz Tota, Tadeusz Ambroży, Kazimierz Witkowski, Tomasz Pałka

**Affiliations:** 1 Institute of Sports Sciences, University of Physical Education, 31-571 Kraków, Poland; 2 Department of Endocrinology, Institute of Sport—National Research Institute, 01-982 Warsaw, Poland; 3 Department of Pathophysiology, Institute of Medical Sciences, Medical College of Rzeszów University, 35-959 Rzeszów, Poland; 4 Faculty of Physical Education and Sports, Wroclaw University of Health and Sport Sciences, 51-612 Wroclaw, Poland; 5 Department of Physiology and Biochemistry, Faculty of Physical Education and Sport, University of Physical Education, 31-571 Kraków, Poland

**Keywords:** Kickboxing, Cortisol, Testosterone, Brain activity

## Abstract

K1-format kickboxing is a widely followed combat sport that requires intense physical exercise. However, research into the body’s response to this type of combat is sparse. This study aims to assess the alterations in hormone levels and brain activity in elite kickboxers following an actual K1 bout and compare these changes with those observed in a control group engaged in a simulated fight exercise with a punchbag. The study included 100 male professional kickboxers, randomly divided into two groups: an experimental group (K1 fight) and a control group (simulated fight with a punchbag). Blood samples were obtained before and after exercise to evaluate testosterone (T) and cortisol concentrations (C). Concurrently, brain activity was recorded using quantitative electroencephalography (QEEG). After the activity in the experimental group mean testosterone level slightly, non-significantly decreased from 13.7 nmol/l to 12.4 nmol/l, while mean cortisol significantly (p < 0.001) increased from 313 to 570 nmol/l. In the control group after the exertion against a punchbag mean cortisol significantly (p < 0.001) increased from 334 to 452 nmol/l and testosterone increased non-significantly, from 15.1 to 16.3 nmol/l. In both groups, the testosterone/cortisol ratio (T/C ratio) showed significantly lower levels after the intervention (p < 0.001 and p < 0.032) in the experimental and control group respectively. The comparison of groups after exercise revealed significantly higher cortisol levels (experimental group x = 570 nmol/l; control group x = 452 nmol/l) and a significantly lower T/C ratio (experimental group x = 2.7; control group x = 3.9), (p = 0.001) in the experimental group. Significantly higher brain activity was found in selected leads after a bout (experimental group). Furthermore, in the experimental group, significant associations of weak to moderate strength were found between hormone fluctuations and selected areas of brain activity (p < 0.05). K1-format kickboxing induces a stress response, evident in the sharp changes in cortisol and testosterone levels. A notable observation was the inverse direction of changes in both hormones. Brain activity analysis indicated the potential influence of raised cortisol concentrations on specific brain areas. This study augments our understanding of the physiological responses during K1 kickboxing bouts and may inform the future evolution of this sport.

## INTRODUCTION

Kickboxing is a spectacular combat sport based on punches and kicks. The sport attracts many spectators who watch sports competitions and practitioners who actively participate in training and competitions at various skill levels [1, 2]. The athletes fight in a standing position and they perform punches and kicks using upper and lower limbs. They are allowed to use full power, in order to demonstrate an advantage over the opponent [3]. The bout can be settled by knockout, technical knockout (inability of the opponent to continue the fight), corner retirement, or judge’s decision (advantage of technical points after the full standard time of the fight) [2]. Importantly, kickboxing requires the use of different motor skills and both aerobic and anaerobic capacity [4].

In training and competing in combat sports, understanding and optimizing adaptive potential is critical [5–7]. Central to the adaptive mechanisms is the endocrine system, with numerous alterations induced by physical exercise. Endogenous testosterone (T) and cortisol (C) levels are frequently tested in elite athletes as hormonal changes are indicative of the body’s response to physical or psychological stimuli [8, 9]. While cortisol facilitates catabolic processes, testosterone fosters anabolic ones. Their balance, reflected in T/C ratio, is crucial [10, 11] and has been studied in sports such as judo [12], wrestling [13], boxing [14], and taekwondo [15]. In kickboxing, differences in cortisol and testosterone concentrations have been found between competitive fighting and experimental forms of training [16]. No hormonal differences between winners and losers have been detected in kickboxing sparring simulations [17]. Slimani et al. reported that integrating mental training for kickboxers resulted in decreased cortisol levels mitigating stress [18].

The intrinsic competitive nature of kickboxing requires intense physical confrontations, making athletes susceptible to multiple injuries [19–21]. Especially concerning is the vulnerability of the head, a topic much discussed in boxing [22]. Post-fight mortality rates and cardiac arrests leading to debilitating and permanent injuries have been documented [23]. The K1 kickboxing format, characterized by its lenient regulations on strike force and the use of knee strikes [3], often witnesses fighters aiming to win the fight by knockout. This illustrates the aspect of the quality of blows delivered with full power. Furthermore, in quantitative terms, careful observation and scientific analysis reveal how a large proportion of strikes is performed directly to the head, or indirectly to the guard in K1 bouts, and qualitative inference has been made in other striking combat sports [24]. In 2007, Tanriverdi et al. established a link between amateur kickboxing and hypopituitarism [25]. It is also known that active and retired athletes alike have demonstrated significant correlations between cognitive functions and GH-IGF-I levels [26]. Follmer et al. emphasized that coaches and athletes need to learn how to recognize concussion symptoms and give first aid [27]. Recent findings suggested brain dysfunctions in K1 fighters that need to be further explored [25, 26]. Currently, electroencephalography (EEG) is popular and widely used in the medical community, providing physicians with important diagnostic information on brain function, with possible neurological disorders. Importantly, EEG is a very practical and inexpensive method of functional neuroimaging [28]. EEG is a qualitative method. On the other hand, the use of quantitative electroencephalography (QEEG), i.e. the analysis of individual brain waves and the ability to extract them, is of increasing interest to researchers in the field of sports [29]. Previous analyses in this area of research indicated that elite athletes had high amplitudes of beta 1 and beta 2 waves in the frontal lobe area during the period of detraining compared to normal values and non-athletes, which, according to the authors, could be related to the accumulation of emotions that negatively affect judgment and coordination [30]. Another study evaluating brain wave activity in kickboxers showed high intensity of sensorimotor rhythm (SMR), beta 1, and beta 2 waves. It was found that high activity of these waves can cause anxiety, stress, difficulty concentrating, and reduced levels of physical activity [31]. Global conclusions from these studies indicate that specific brain wave activity can have both negative and positive effects on brain function and indirectly on sports performance. In contrast, in a state of immediate combat readiness, before a sporting event, fighters exhibit elevated and diverse brain wave activity, associated with stress, arousal, and concentration [32]. Participating in this sport might lead to severe and irreversible neurological conditions.

K1 kickboxing offers opportunities to analyse the body’s neuroendocrine and neurophysiological responses to stress, with athletes enduring rigorous physical strain, psychological exposure, and potentially serious injuries. Notwithstanding the burgeoning interest [6], literature surveys have shown a paucity of comprehensive studies integrating hormonal, neurophysiological, and neurocognitive analyses during K1 bouts. This research gap highlights the need for holistic diagnosis in this domain.

This study pursues two primary objectives:

1. To investigate hormonal and post-bout alterations in brain activity in K1 kickboxers (experimental group) compared to a control group participating in a punchbag session simulating fight conditions.
2. To evaluate relationships between hormonal profiles and brain activity.

The findings here can enhance our understanding of neuroendocrine dynamics during kickboxing, thus providing a foundation for therapeutic strategies and innovative corrective therapies.

In our study, based on previous scientific reports, we hypothesized that depending on the type of stimulus (real fight or punchbag), there would be variation in the levels of the hormone indices studied.

## MATERIALS AND METHODS

### Participants

The study involved 100 male professional kickboxers from Poland, all competing in the K1 format. The mean age (years), body height (cm), and body mass (kg) of participants in the research population were 26.06 ± 4.9 (range: 18–35), 178.5 ± 5.9 (range 165–189), and 77.3 ± 8.8 (range: 61.4–94.1) respectively. Inclusion criteria were as follows: a minimum of 5 years of training experience, absence of injuries, up-to-date medical examinations, no history of severe knockouts, a positive medical recommendation, and active participation in competitions. Exclusion criteria included having less than 5 years of training experience, conditions such as seizures or other neurological disorders, presence of injuries, lack of participation in competitions, history of severe knockouts, or a negative medical recommendation. The athletes were randomly divided into two groups: the control group (n = 50) and the experimental group (n = 50). The mean age (years), body height (cm), and body mass (kg) of participants in the experimental group were 25.5 ± 4.6 (range: 18–33), 179.3 ± 6.1 (range 168–189), and 76.7 ± 10.0 (range: 61.4–94.1) respectively. The mean age (years), body height (cm), and body mas (kg) of participants in the control group were 26.6 ± 5.2 (range: 18–35), 177.7 ± 5.6 (range 165–187), and 77.9 ± 7.5 (range: 65.7–90.0) respectively. The sample size was determined using G*power (confidence level 95%, margin of error 5%). Each participant was assigned a unique identification number, and group placement was determined through a random number generator. All participants were informed in detail about the experimental procedure and provided written consent to participate in the experiment. The study was conducted according to the Declaration of Helsinki and approved by the Ethics Committee of the University of Rzeszów (protocol code 8/12/2021).

### Methods

The athletes in the experimental group participated in simulated sparring in the ring, organized for this research project according to the K1 rules adopted by the World Association of Kickboxing Organizations (WAKO) [33]. The bout was divided into 3 rounds lasting 2 minutes each, with a one-minute break between rounds. The athletes were divided by weight categories. The bouts were supervised by a qualified and licensed judge. Twenty-five bouts were held over the full duration of standard fighting time. The study of both groups was conducted before noon. The control group athletes participated in a punchbag training session that simulated the conditions of a kickboxing match. The fatigue-focused session consisted of three 2-minute work periods (equivalent to K1 rounds) separated by a oneminute break between them. The participant stood in a fighting stance, directly in front of the punchbag at a fighting distance. During each period of work, following the command *Start*, the participant performed as many offensive combinations of K1 formula techniques as possible, performed individually or in series. The focus was on an intense effort. The command *Stop* marked the end of a given work period and a one-minute break after the first and second work periods. The simulation was developed on request. In order to confirm the relevance of the intervention, participants were measured for cardiovascular stress parameters (HR $\hat{\text{x}}$ = 182.67 ± 5.9), which indicated that it represented a test of very high-intensity exercise compared to a sports bout. The results obtained are similar to literature data indicating that in trained athletes, intense exercise based on hand-to-hand combat techniques results in an increase in the minute heart rate ranging from 165 to 185 beats per minute [34]. Blood samples (1.5 ml each) were collected from the antecubital vein of participants from both groups before the warm-up and 3 minutes after exercise. These procedures were performed by a qualified nurse. Serum concentrations of testosterone and cortisol were evaluated based on the electrochemiluminescence method, using a COBAS Integra 400 Plus analyser. Every batch of analysis included control sera to gauge the precision of the measurements. The within-series coefficient of variation (CV) for both hormones was 3.8%, and the between-series CV was 4.2%. On the basis of T and C concentration values, the anabolic-catabolic balance index was calculated (T/C * 100).

### QEEG Analysis

The QEEG method was used to identify the profile of brain wave activity in the kickboxers before and after the experimental intervention and to diagnose the relationship with hormonal pattern. Quantitative electroencephalography (QEEG) offers a numerical spectral analysis of the EEG record. This process involves digitally coding the data, followed by a statistical analysis using the Fourier transform algorithm. Each examination, conducted individually, lasted roughly 10–15 minutes and comprised two phases: recording brainwave activity with eyes closed (3 min) and with eyes open (3 min). The amplitude and power of distinct frequencies were scrutinized. Based on established norms for adults, a general rule was that the lower the wave frequency was, the higher was its amplitude. Established standard values were delta waves under 20 µV, theta under 15 µV, alpha under 10 µV, and SMR, beta 1, and beta 2 in the range 4–10 µV. The EEG signal was processed using a Cz montage with the Cz electrode often employed as the primary reference. Quantification was performed using Elmiko DigiTrack software (version 15 PL, ELMIKO, Poland). Delta, theta, alpha, SMR, beta 1, and beta 2 waves were recorded at nine electrode points: frontal (FzF3F4), central (CzC3C4), and parietal (PzP3P4). The amplitude of QEEG rhythm met the medical standards specified for the DigiTrack device. A signal spectrum reflects its frequency-dependent pattern. The fast Fourier transform (FFT algorithm) was employed, yielding the function: f(z) = A(z) + j*F(z). FFT analysis utilized parameters such as a minimum signal amplitude of 0.5 µV and a minimum temporal gap between individual peak values of 0.5 Hz. Analysis was performed with a computation buffer of 8.2 s (2048 assessment points, accuracy of 0.12 Hz). Consequently, an amplitude value set for each segment of the frequency spectrum was generated. The gap between individual values, measured in Hz, determined the computation resolution. For the FFT algorithm, this parameter depends on the signal sampling frequency and the computation buffer length: r = fs/N, where ‘r’ is the computation resolution, ‘fs’ is the signal sampling frequency, and ‘N’ denotes the computation buffer length. The FFT panel in DigiTrack displayed peak-to-peak amplitudes. Measurement epochs of several seconds were used to ensure reliable results. The duration of these epochs determined the frequency resolution of the Fourier transform: a 1-second epoch translated into a 1 Hz resolution (± 0.5 Hz), while a 4-second epoch yielded 0.25 Hz or ± 0.125 Hz resolution. Artefact removal from the EEG recording was performed both manually and automatically. For the experimental group, the initial test was executed before the warm-up, and the subsequent test immediately after the K1 kickboxing match. Each bout consisted of three 2-minute rounds interspersed with one-minute intervals. For the control group, the baseline measurement was performed before the warm-up, and the next one followed a simulated bout with a punchbag, structured identically to that in the experimental group [30, 31, 35, 36]. The results of the QEEG for open eyes are presented in this study since only these values showed statistically significant correlations.

### Statistical Analysis Methods

In the study’s data analysis, basic statistical methods were employed, including the calculation of the arithmetic mean and standard deviation. Additionally, specialized statistical analyses were conducted, such as relative to variables and the differentiation in brain activity of the experimental group, diagnosed before and after intervention. A one-way analysis of variance (ANOVA) with repeated measures and a post-hoc Bonferroni test were used. For assessing the variation in hormonal indicators, before and after the intervention in the studied groups, a two-way analysis of variance with repeated measures (group * time) was utilized. Effect sizes for each time and group factor were determined, followed by the application of the post-hoc Bonferroni test for comparing variables between measurements and between groups using an interaction layout. Linear correlation coefficients were employed to evaluate the relationships between variables (brain activity vs. hormonal indicators). The inference thresholds for Pearson’s correlation are as follows: 0.50–1.00 – strong correlation, 0.30–0.49 – moderate correlation, below 0.29 – low correlation [37]. All data analyses were conducted using STATISTICA software version 13.3. Statistical significance was established at a p-value of less than 0.05.

## RESULTS

Table 1 presents the statistical characteristics of the studied hormonal indicators before and after the research intervention in both groups, along with the results of the two-factor analysis of variance. Intragroup comparison revealed a significant increase in post-exertion cortisol concentrations in both research groups. An opposite trend was observed regarding the T-to-C ratio. A significant decrease in the value of the anabolic-catabolic index (T/C*100) was observed in both research groups (Table 1). The post-exertion relative increase in cortisol concentration in the experimental group (by 82%) was over twice as high as that in the control group (by 35%). The value of the anabolic-catabolic index decreased by 2.2 points in the experimental group (1.1 points in the control group). No significant post-exertion changes in testosterone concentration were observed in either group, but there was a slight decrease in this hormone’s level in the experimental group by 9.5% and an increase in the control group (by 7.9%).

**TABLE 1:** Descriptive statistics and results of the two-factor analysis of variance for hormonal parameters in the control and experimental groups.

Variable	EXP		CON		Between groups		interaction Variable*time	

T (nmol/l)	Mean	SD	Mean	SD				
Pre	13.7	2.7	15.1	3.9	p = 0.309	p	Partial η^2^	power

Post	12.4	2.4	16.3	5.0	**p < 0.001**			

Between examination	p = 0.456		p = 0.641			**< 0.017**	**0.029**	**0.666**

Time effect	p = 0.907 partial η^2^ < 0.001 power = 0.052							

**C (nmol/l)**	**EXP**		**CON**		**Between groups**		interaction Variable*time	

	**Mean**	**SD**	**Mean**	**SD**				

Pre	312.9	95.1	334.2	82.4	p = 1.000	p	Partial η2	power

Post	570.4	123.9	451.8	94.1	**p < 0.001**			

Between examinations	**p < 0.001**		**p < 0.001**			**< 0.001**	**0. 111**	**0.998**

Time effect	**p < 0.001 partial η^2^ = 0.473 power = 1.000**							

T/C*100	**EXP**		**CON**		**Between groups**		interaction Variable*time	

	**Mean**	**SD**	**Mean**	**SD**				

Pre	4.9	2.2	5.0	2.8	p = 0.073	p	Partial η^2^	power

Post	2.7	1.0	3.9	1.9	**p = 0.001**			

Between examinations	**p < 0.001**		**p = 0.032**			**< 0.015**	**0.030**	**0.683**

Time effect	**p < 0.001. partial η^2^ = 0.174 power = 0.999**							

EXP – Experimental Group, Con – Control Group, T-Testosterone, C – Cortisol, SD – Standard Devotion, p – significance

Inter-group comparisons revealed no significant differences for testosterone, cortisol, and the anabolic-catabolic index (T/C*100) before exertion. Following the application of the intervention stimulus, statistically significant intergroup differences in the studied hormonal parameters were found. In the experimental group, the C level was higher (by 20.8%), and lower values for T (by 31.5%) and T/C*100 (by 44.4%) were noted compared to the corresponding parameters for the control group (Table 1).

Table 2 shows the nature of the co-occurrence between selected hormone parameters and parameters of brain electrical activity (QEEG), after the K1 bout in the experimental group. There was a significant negative correlation between alpha frequency (Fz) and T of moderate strength. A similar trend was noted between Beta 1 (P3) and C, with an inverse positive direction of correlation. Significant and weak positive correlations were found between alpha (P3), SMR (F3), beta 1 (F3), and C. Theta (C4) and alpha (Fz) showed a similar profile of correlations with T/C, with a different (negative) direction (see Table 2). In the experimental group, there was a moderate yet close to the lower limit of strong negative correlation between cortisol and testosterone levels after the K1 bout (r = -0.47; p < 0.05). No such correlations were found in the control group or the two study groups before exercise.

**TABLE 2:** Correlations between testosterone levels, cortisol levels, the testosterone/cortisol ratio, and QEEG parameters after the fight in the experimental group.

Parameter	Testosterone, (nmol/l)		Cortisol, (nmol/l)		Testosterone/Cortisol	

	r	p	r	p	r	p
Theta 4 Hz-8 Hz (C4)	-0.19	0.180	0.23	0.102	-0.28*	**0.048**
Alpha 8 Hz-12 Hz (Fz)	-0.30**	**0.034**	0.15	0.303	-0.29*	**0.043**
Alpha 8 Hz-12 Hz (P3)	0.12	0.404	0.29*	**0.038**	-0.09	0.513
SMR 12 Hz-15 Hz (F3)	0.04	0.806	0.29*	**0.040**	-0.10	0.481
Beta1 15 Hz-20 Hz (F3)	0.15	0.310	0.29*	**0.039**	-0.05	0.715
Beta1 15 Hz-20 Hz (P3)	0.17	0.237	0.30**	**0.034**	-0.05	0.717

* – low correlation,

** – moderate correlation, C4 – right central electrode, Fz – central frontal electrode, P3 – left occipital electrode, F3 – left frontal electrode

Table 3 displays the average values (for selected leads) of brain electrical activity before and after the research intervention in the experimental group, along with the results of the comparative analysis. The presented parameters played a significant role in the previously described significant relationships (Table 2). The comparison of means showed statistically significant differences for all variables, where their activity markedly increased. The exception was the alpha wave (lead Fz), whose amplitude increased slightly by 4.5%.

**TABLE 3:** Results of One-way ANOVA and post-hoc Bonferroni test for selected QEEG electrodes (which demonstrated statistically significant correlations) before and after the intervention in the experimental group.

Brain waves parameters	Pre intervention	Post intervention	p-value	Partial η^2^	power
Theta 4 Hz-8 Hz (C4)	6.64 ± 1.21	7.21 ± 1.26	**0.016**	**0.058**	**0.680**
Alpha 8 Hz-12 Hz (Fz)	7.26 ± 1.75	7.60 ± 1.76	0.333	0.009	0.161
Alpha 8 Hz-12 Hz (P3)	7.75 ± 2.54	9.35 ± 3.32	**0.014**	**0.060**	**0.696**
SMR 12 Hz-15 Hz (F3)	4.39 ± 0.73	5.94 ± 1.14	**< 0.001**	**0.396**	**1.000**
Beta1 15 Hz-20 Hz (F3)	5.50 ± 0.65	6.54 ± 1.24	**< 0.001**	**0.191**	**0.997**
Beta1 15 Hz-20 Hz (P3)	5.53 ± 0.72	6.46 ± 1.06	**< 0.001**	**0.256**	**0.999**

C4 – right central electrode, Fz – central frontal electrode, P3 – left occipital electrode, F3 – left frontal electrode

## DISCUSSION

Cortisol and testosterone responses to exercise depending on its duration and intensity have been extensively described in the literature. Prolonged exercise, such as long-distance running [38–41] or triathlon competitions [42], often leads to a significant increase in cortisol levels and a decrease in testosterone levels. However, after shorter maximal exercise, testosterone levels do not decrease and they often increase, as do levels of luteinizing hormone (LH) [43, 44]. This suggests the stimulation of the hypothalamic-pituitary-gonadal (HPG) axis. Evaluation of post-exercise testosterone changes should focus not only on secretion but also on transient blood concentration. This nuance can obscure the testosterone changes induced by secretion, and researchers sometimes neglect this in their analyses. If the focus is solely on immediate post-exercise androgenic activity in the blood without considering the causes of changes in androgen concentration, the post-exercise haemoconcentration effect can be missed. This effect can rise to several percent directly after supramaximal exercise [45].

Although a single competitive effort in combat sports lasts several minutes, it intensely activates both the HPA (hypothalamic-pituitary-adrenal) and HPG (hypothalamic-pituitary-gonadal) axes, elevating both cortisol and testosterone levels. However, contrary to that rule, in our study, we observed a rise in cortisol only but not in the androgen level after a 3-round fight. The control group, performing exercise of similar duration and intensity with a punchbag, showed a smaller hormone increase, likely due to the absence or minimal psycho-emotional component of the post-exercise stress. Although this group performed a simulated fight, they did not receive any blows. Other studies have also reported elevated cortisol levels following a real fight compared to contact-free exercise simulating a kickboxing bout [16]. The consistent cortisol levels between groups before the exercise suggest only minimal HPA axis activation before the fight, which was not as demanding as official competitions. Especially noteworthy is the observed interaction between the two hormones after the bout in the experimental group. A negative correlation was found between cortisol and testosterone levels, due to the inhibitory effect of elevated cortisol levels on the HPG axis in men. This phenomenon occurs either when cortisol levels are particularly high or for longer exposure durations, as corroborated by other studies [8, 38–42, 46]. In the post-exercise recovery period following two sequential exhaustive workouts, a significant negative correlation between testosterone and cortisol was also observed [43].

In evaluating the physiological and metabolic changes induced by cortisol, it is necessary to emphasize that most effects are attributed to the unbound cortisol fraction. This fraction, considered biologically active, diffuses from the circulation into target cells and comprises roughly 10–15% of the total hormone concentration. As total cortisol concentration increases, the unbound fraction does as well but not proportionally. A marked increase in free cortisol becomes evident when the total hormone concentration exceeds 500 nmol/l [44]. In our study, only 13 athletes from the control group and 30 from the experimental group had post-exercise levels above 500 nmol/l. Perhaps the interaction between the hormones and the shifts in brain electrical activity after a kickboxing match are more influenced by the elevated concentration of the biologically active fraction that permeates the blood-brain barrier than by the cumulative cortisol concentration. This theory is consistent with the observed positive post-bout correlations between cortisol and alpha 8–12 Hz (P3), beta1 15–20 Hz (F3), and beta1 15–20 Hz (P3) frequencies in the experimental group. These findings are similar to those described in a review article [45], suggesting the possibility that either endogenous or exogenous cortisol stimulates regions of the limbic system, including the amygdala, hippocampus, paraventricular nucleus, and prefrontal cortex, and that shifts in delta and beta wave amplitudes reflect the degree of stress induced by perceived threats [47, 48]. Brain activity, especially in the pituitary-hypothalamic area, can affect the regulation of sex hormones, including testosterone. The brain controls the secretion of hormones by the pituitary gland, which in turn controls the secretion of hormones by the testes in men, including testosterone [49]. Stress, poor nutrition, sleep deprivation, and other factors can affect the functioning of the endocrine system, testosterone levels, and thus the brain waves. A variety of meditation practices used by athletes before a fight, associated with alpha waves, can affect the body’s hormonal balance, including levels of sex hormones such as testosterone. Exercises related to relaxation, meditation, and breathing techniques help athletes reduce stress and tension. In a state of relaxation, an increase in alpha waves in the brain can be observed [50].

Available research also suggests that reducing stress levels and stimulating alpha wave activity can affect overall hormonal balance, including levels of sex hormones such as testosterone. Reduced stress levels can help maintain hormonal balance. Sleep is a key part of recovery, including hormonal recovery. Theta waves are also associated with a certain stage of sleep. If sleep is disturbed, a person experiences fluctuations within this wave, followed by fluctuations in hormone levels. Sleep deprivation can lead to lower testosterone levels. There is a link between cortisol levels and brain wave activity, especially in terms of the body’s response to stress [51]. During stressful situations, cortisol levels in the body increase. Cortisol plays an important role in the stress response, helping the body adapt to difficult situations. It affects many body functions, including brain function. Brain wave activity can change in response to stress. For example, in stressful situations, there may be an increase in beta wave frequencies, which is associated with a more alert state of mind. In general, there is a complex interaction between cortisol and brainwave activity, but this depends on many factors, including the type of stress, the duration of the stress response, and individual variability [52]. Research in this field is still ongoing, and understanding this interaction may contribute to a better understanding of the body’s response to stress and its impact on brain function.

Addressing the influence of kickboxing on brain electrical activity mandates consideration of EEG study results linked to laboratory exercises devoid of head injury risk. Increases in alpha, theta, beta, and gamma wave amplitudes following strength training have been documented [53]. During progressively intense cycling on an ergometer, alpha, theta, and beta wave activity across various brain regions seemed to increase simultaneously [54]. Moreover, the extent of postexercise EEG alterations depends on individual exercise preferences [55]. Another investigation revealed that after acclimatizing to exercise over a training period, the peak frequency of alpha waves rose only following exhaustive maximal exercise rather than moderate, stable-intensity exercise without emotional arousal [56]. A few studies have concentrated on brain electrical activity in relation to androgen status. It was reported that exogenous testosterone amplified affective stress responses, notably in dominant males [57]. Interpreting the negative correlation between alpha 8–12 Hz (Fz) and endogenous testosterone is difficult without the analysis of bout outcomes and psychological studies. This underlines the necessity for further more comprehensive kickboxing research that includes psychological assessment [58].

### Limitations of the Study

The lack of research opportunities and activities regarding the diagnosis related to tournament fights means that the results of this study may be limited to a certain extent. However, in light of the inevitable limitations of our experimental design, we recruited a significant number of elite K1 athletes, the procedures were carried out according to a strict protocol, and the results are scientifically valid. To capture the multifaceted clinical context, future research should consider making comparisons between other populations, such as athletes of other combat sports, non-athletes, and groups of female athletes.

## CONCLUSIONS

1. Both study groups showed significant psychophysiological stress following the application of the experimental stimulus, resulting in strong activation of the HPA axis and noticeable changes in blood testosterone levels.
2. Post-intervention comparative analysis showed significantly higher cortisol levels and a significantly lower T/C ratio for the experimental group performing real bouts.
3. When confronted with an opponent (simulated sparring), there was a significantly negative correlation between cortisol and testosterone levels in the experimental group.
4. Simulated K1 sparring in the experimental group resulted in a significant increase in brainwave activity.
5. In the immediate recovery phase for the experimental group, there were statistically significant correlations between cortisol and selected indices of brain electrical activity. Such associations did not occur after equivalent non-contact exercise.

### Practical Implications

The results of the study inspire further research on the relationship of a kickboxing bout with selected brain bioelectrical indices, hormonal activity, and hormonal-neurophysiological interactions in athletes. The results of these observations can also affect the programming of the training process and significantly affect the modification of regulations in combat sports, emphasizing the safety of participants and enhancing health protection measures.
